# Screening for the Prevalence of Nonalcoholic Fatty Liver Disease (NAFLD) Among Patients With Prediabetes and Type 2 Diabetes: A Comparison of Three Screening Systems

**DOI:** 10.1155/ije/6676114

**Published:** 2025-08-21

**Authors:** Ruba H. Alhabahbeh, Ala'eddien N. Obeidat, Dunia S. Jaber, Mohammed M. AlKhaldi, Leen K. Ghanem, Ahmad A. Tubasi, Zaina N. Obeidat, Hussam H. Alhawari

**Affiliations:** ^1^Department of Family Medicine and Public Health, School of Medicine, University of Jordan, Amman, Jordan; ^2^Department of Internal Medicine, School of Medicine, University of Jordan, Amman, Jordan

**Keywords:** diabetes, liver fibrosis, nonalcoholic fatty liver disease (NAFLD), prediabetes, screening systems

## Abstract

**Purpose:** To screen for the prevalence and severity of nonalcoholic fatty liver disease (NAFLD) and degree of liver fibrosis in patients with prediabetes and Type 2 diabetes. Additionally, we sought to compare the results obtained from different screening systems.

**Methods:** We screened 254 patients for NAFLD using three systems: Fatty Liver Index (FLI), Fibrosis 4 (FIB-4) Index, and NAFLD Fibrosis Score (NFS). About two-thirds were females (63%). The mean age was 59.15 ± 10.09 years, and mean BMI was 34.14 ± 6.60 kg/m^2^. Among participants, 85.5% had Type 2 diabetes and 14.5% had prediabetes. Additionally, 81.1% were on metformin, and 39.6% were on insulin.

**Results:** Probable steatosis (NAFLD) prevalence was 77.0% (FLI score) in our cohort. Moderate to advanced liver fibrosis was 12.6% (NFS score) and 20.7% (FIB-4 score). Significant discrepancies were noted: FIB-4 identified 21.6% of patients with moderate to severe fibrosis, which FLI did not recognize as NAFLD. FIB-4 also identified 26 patients with moderate to severe fibrosis that NFS missed. The FIB-4 and FLI score discrepancy was more common in females (10.2% vs. 1.7%, *p* = 0.046) and in patients with diabetes compared to prediabetes (21.7% vs. 4.5%, *p* = 0.003). The FIB-4 and NFS score discrepancy was more common in patients with higher BMI (38.38 ± 7.78 vs. 33.59 ± 6.82, *p* < 0.001) and in those with prediabetes compared to diabetes (34.8% vs. 12.8%, *p* = 0.008).

**Conclusion:** The study found a high prevalence (77%) of NAFLD in individuals with prediabetes and diabetes. About 20% had moderate to advanced liver fibrosis. NAFLD prevalence and severity varied significantly across three scoring systems. Key factors for refining screening strategies include patient sex, BMI, and the level of insulin resistance.

## 1. Introduction

Diabetes and liver disease are closely linked, with about 70% of patients with Type 2 diabetes (T2D) having associated liver disease, predominantly nonalcoholic fatty liver disease (NAFLD) [1], or what has been recently termed metabolic-associated fatty liver disease (MAFLD) in some literature [2]. NAFLD can range in severity from simple excessive fat accumulation within the liver (simple steatosis) to hepatic inflammation (nonalcoholic steatohepatitis) and can progress to varying degrees of liver fibrosis, cirrhosis, and end-stage liver disease (ESLD) [3]. The principal risk factors for NAFLD include increased Body Mass Index (BMI), encompassing overweight and obesity, and T2D [3].

The American Diabetes Association (ADA) recently updated its screening guidelines on June 24th, 2023, and now advises universal screening for NAFLD in individuals with T2D and prediabetes using the calculated Fibrosis 4 (FIB-4) Index, which is derived from age, aspartate aminotransferase (AST), alanine aminotransferase (ALT), and platelet count [4]. Interestingly, the ADA has chosen to retain the nomenclature of fatty liver disease as NAFLD rather than updating it to MAFLD [4]. Accordingly, we have adhered to the ADA's preference in our manuscript.

In our study, we evaluated the prevalence of NAFLD, including moderate to severe liver fibrosis, among patients with T2D and prediabetes using the calculated FIB-4 index [4]. Additionally, we compared the sensitivity of the FIB-4 score with the Nonalcoholic Fatty Liver Disease Fibrosis Score (NFS) and assessed the impact of patient variables on these screening scores. Furthermore, we employed the Fatty Liver Index (FLI) as a general screening tool for NAFLD prevalence, irrespective of severity.

## 2. Materials and Methods

### 2.1. Subjects

This single-center study was conducted from December 2023 to June 2024 at the Endocrinology Clinic of Jordan University Hospital (JUH), a tertiary medical center in Amman, Jordan. The study protocol was approved by the Institutional Review Board (IRB) at JUH (IRB approval number: 10 2023/30524). Prior to participation, informed consent was obtained from all participants involved in the study.

Subjects diagnosed with prediabetes and T2D were recruited from patients attending the Endocrinology Clinic at JUH during the study period. Inclusion criteria comprised males or females aged 16 to 65 years, diagnosed with T2D or prediabetes based on the latest ADA guidelines in 2023 [4]. To ensure the accuracy of screening, patients already diagnosed with NAFLD or other liver disorders were excluded. Most participants were females (63.0%). The mean age and BMI were 59.15 ± 10.09 years and 34.14 ± 6.60 kg/m^2^, respectively. Among the participants, 218 (85.5%) had T2D, while the remaining 14.5% had prediabetes.

We screened 254 patients to assess the prevalence of fatty liver disease and liver fibrosis using three screening systems: (1) the FIB-4 Index, (2) NFS, and (3) FLI.

### 2.2. Assessments

Data on age, gender, BMI (BMI =Weight in kilograms/(Height in meters)^2^, waist circumference (WC) in centimeters, and duration of diabetes were recorded for each participant. Serum AST, ALT, and γ-glutamyltransferase (GGT) levels were quantitatively determined using the Roche cobas c system. Normal reference ranges were established as follows: AST 8–33 units per liter (U/L), ALT 7–56 U/L, and GGT 5–40 U/L (Roche Diagnostics GmbH; 2020). Serum triglyceride levels were quantitatively determined using the Roche cobas c system, with a normal triglyceride level considered below 150 mg/dL (Roche Diagnostics GmbH; 2020). Serum albumin levels were quantitatively determined using the Roche cobas c system, with a normal range of 3.4–5.4 g per deciliter (g/dL) (Roche Diagnostics GmbH; 2020).

The FIB-4 Index assesses the presence of advanced liver fibrosis, calculated from age, AST, ALT, and platelet count [4, 5]. The formula for calculating FIB-4 is “FIB-4 = Age ([yr] × AST [U/L])/((PLT [10(9)/L]) × (ALT [U/L]) (1/2)” [5]. Interpretation of the FIB-4 Score in relation to advanced liver fibrosis risk is as follows: A score of < 1.30 indicates low risk, scores between 1.30 and 2.67 indicate intermediate risk, and a score of > 2.67 indicates high risk for advanced fibrosis [6].

The NFS evaluates the presence of advanced liver fibrosis, calculated from age, AST, ALT, platelet count, BMI, serum albumin, and the presence of prediabetes or diabetes [7]. The formula for calculating NFS is “NFS = −1.675 + 0.037 × age (years) + 0.094 × BMI (kg/m^2^) + 1.13 × (impaired fasting glycemia or diabetes [yes = 1, no = 0]) + 0.99 ×  (AST/ALT ratio) − 0.013 × platelets (× 10^9^/L) − 0.66 × albumin (g/dL)” [8]. Interpretation of the NFS in relation to significant liver fibrosis is as follows: A score of < −1.455 indicates no significant fibrosis (F0–F2 fibrosis), a score > 0.675 indicates the presence of significant fibrosis (F3–F4 fibrosis), and a score between −1.455 and 0.675 is considered indeterminate [8].

The FLI determines the presence or absence of probable steatosis (NAFLD), calculated from BMI, WC, serum triglycerides, and serum GGT [9]. The formula for calculating FLI is “FLI = ey/(1 + ey) × 100, where: *y* = 0.953 × triglycerides (TGs) (mg/dL) + 0.139 × BMI (kg/m^2^) + 0.718 × GGT(U/L) + 0.053 × WC (cm) − 15,745” [10]. Interpretation of the FLI score, which ranges from 0 to 100, in relation to the presence of NAFLD is as follows: An FLI < 20 rules out the presence of steatosis, a score of ≥ 60 suggests the presence of probable steatosis, and a score between 20 and 60 is considered indeterminate [10].

### 2.3. Statistical Analysis

IBM SPSS Statistics Version 24 was utilized for data analysis. Continuous variables were summarized using mean and standard deviation, while categorical variables were presented as counts and percentages. Initially, the overall prevalence of NAFLD was determined based on the FLI score. Subsequently, the extent of fibrosis, categorized as “none to mild” and “moderate to severe,” was assessed using the FIB-4 and NFS.

To examine the association between patient characteristics and moderate to severe fibrosis using the FIB-4 and NFS metrics, *T*-tests and chi-square tests were employed. Additionally, the percentage of disagreement between these two metrics was calculated. Factors contributing to this discrepancy were further explored using *T*-tests and chi-square tests.

A significance level of *p* < 0.050 was considered statistically significant for all analyses.

## 3. Results

### 3.1. Patients' Characteristics

A total of 254 patients were included in our study. Majority of patients were females (63.0%). The mean age and BMI were 59.15 ± 10.09 and 34.14 ± 6.60, respectively. The number of patients with T2D was 218 (85.5%), while the rest had prediabetes (14.5%). Additionally, 81.1% of the patients were on metformin, while 39.6% were on insulin.  Table 1 displays the characteristics of the included patients.

### 3.2. Factors Associated With Fibrosis Diagnosed Using Different Scoring Systems

**Figure 1:**
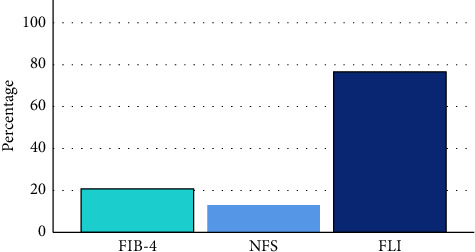
The percentage of cases with advanced liver fibrosis in FIB-4 and NFS. The percentage of cases with probable steatosis (NAFLD) in FLI score.

In our cohort, the prevalence of advanced liver fibrosis was 20.7% and 12.6% according to the FIB-4 Score and NFS, respectively, while the overall prevalence of probable steatosis (NAFLD) was 77.0% based on the FLI score (Figure 1).

Regarding factors associated with fibrosis assessed by FIB-4, patients with moderate to severe fibrosis were significantly older (67.36 ± 9.44 years) compared to those with no or mild fibrosis (56.72 ± 9.11 years) (*p* < 0.001). Additionally, patients with moderate to severe fibrosis had a longer duration of diabetes, which was statistically significant (*p*=0.046) (Table 2). The total number of patients in Table 2 is 241 because the rest of the study participants did not have their platelet count recorded in their medical records, so we were unable to calculate the FIB-4 Index for them [5].

According to NFS criteria, patients with moderate to severe fibrosis (68.06 ± 8.21 years) were significantly older compared to those with no or mild fibrosis (51.33 ± 8.18 years) (*p* < 0.001). Additionally, BMI and WC were significantly higher in patients with moderate to severe fibrosis compared to those with no or mild fibrosis (*p* < 0.001). Similarly, patients with moderate to severe fibrosis had a longer duration of diabetes, which was statistically significant (*p* < 0.001) (Table 3).

Patients with probable steatosis, as determined by FLI, were less likely to be on metformin (27.4%) compared to those with no steatosis (77.3%) (*p* < 0.001). Furthermore, patients with probable steatosis had higher BMI and WC compared to those with no steatosis (BMI: *p*=0.006, WC: *p*=0.001) (Table 4).

### 3.3. Discrepancies Between the Scoring Systems and Factors Associated

Regarding discrepancies between the scoring systems, FIB-4 identified 11 patients (21.6%) with moderate to severe fibrosis who were not identified as having probable steatosis (NAFLD) by FLI. Conversely, FIB-4 identified 26 patients with moderate to severe fibrosis that NFS did not identify (Table 5).

Several factors were associated with discrepancies between FIB-4 and other scoring systems. Specifically, regarding the discrepancy between FIB-4 and FLI, it was more prevalent among females (10.2%) compared to males (1.7%) (*p*=0.046). Furthermore, this discrepancy was more common among patients with diabetes compared to those with prediabetes (21.7% vs. 4.5%) (*p*=0.003).

The discrepancy between FIB-4 and NFS was more pronounced among patients with higher BMI (38.38 ± 7.78 vs. 33.59 ± 6.82) (*p* < 0.001). Additionally, it was more prevalent among patients with prediabetes compared to those with diabetes (34.8% vs. 12.8%) (*p*=0.008) (Table 6).

## 4. Discussion

Type 2 diabetes mellitus (T2DM) and NAFLD represent significant and escalating global public health challenges [11]. The increasing prevalence of NAFLD mirrors the worldwide rise in obesity, insulin resistance, and diabetes [12]. NAFLD manifests as a spectrum of liver abnormalities, progressing from simple steatosis (excessive fat accumulation in the liver) to nonalcoholic steatohepatitis (hepatic inflammation), and varying degrees of liver fibrosis, cirrhosis, and ESLD [3, 12].

Importantly, NAFLD has emerged as a notable precursor to hepatocellular carcinoma (HCC). Unlike HCC arising from chronic viral infections, approximately 50% of HCC cases associated with NAFLD develop in the absence of cirrhosis, likely due to chronic inflammation induced by hepatic lipotoxicity [13, 14].

Early diagnosis and lifestyle interventions remain the cornerstone of NAFLD management. While approved treatments for NAFLD are currently lacking, several pharmaceutical interventions are under investigation [15–19]. Recent studies have demonstrated the benefits of glucagon-like Peptide 1 receptor (GLP-1R) agonist therapy in NAFLD management [15, 16]. Additionally, sodium-glucose cotransporter Type 2 inhibitors (SGLT-2i) have shown potential benefits in treating NAFLD [17]. A systematic review by Mantovani et al., published in The Lancet Gastroenterology and Hepatology, evaluated three classes of diabetes drugs—peroxisome proliferator-activated receptor (PPAR) agonists, GLP-1R agonists, and SGLT-2 inhibitors—all of which demonstrated potential benefits in NAFLD treatment [18]. The review highlighted improvements with pioglitazone and lanifibranor (PPAR agonists), as well as with liraglutide and semaglutide (GLP-1R agonists), showing improvements in hepatic steatosis and lobular inflammation, and in some cases, resolution of NAFLD without exacerbating fibrosis [18]. Furthermore, SGLT-2 inhibitors were noted for their ability to reduce liver fat content [18].

There are several screening systems available for NAFLD, each with variable sensitivity and limitations; however, they are considered cost-effective and noninvasive tests for screening liver fibrosis in high-risk patients who may benefit from early detection and intervention [19, 20]. Previous systematic reviews and meta-analyses have compared these scoring systems, yielding varied results influenced by their cutoff points, patient age, and sex [21, 22]. Among these screening systems are the FLI score, which assesses the presence of NAFLD irrespective of severity [9, 10], and the FIB-4 and NFS scoring systems, which not only detect NAFLD but also evaluate the extent of liver fibrosis [6–8].

The prevalence of NAFLD in our cohort was 77.0%, as determined by the FLI score, which is slightly higher than the reported prevalence of liver disease of approximately 70% in patients with diabetes [1]. Interestingly, the prevalence of advanced liver fibrosis in our cohort was 20.7% and 12.6% according to FIB-4 Score and NFS, respectively. This indicates that approximately 20% of our participants had undiagnosed moderate to advanced liver fibrosis, highlighting the need for referral to a gastroenterology clinic for further evaluation.

The study identified several factors that influenced the outcomes of the three scoring systems, leading to significant discrepancies. These factors included patient sex, BMI, and the degree of insulin resistance (diabetes vs. prediabetes).

### 4.1. Limitations

Our study has several limitations. Firstly, the absence of confirmatory tests such as liver biopsy or fibroscan may raise concerns about the accuracy of our findings. However, our primary goal was to estimate the prevalence of NAFLD using noninvasive, readily available screening tools in a real-world clinical setting. This approach is particularly relevant for routine clinical practice, where such diagnostic tools are often preferred due to their accessibility and lower cost. A second limitation is the exclusion of patients with a prior diagnosis of liver disease or NAFLD. This decision was made to focus on undiagnosed individuals with prediabetes and T2D, aiming to uncover the hidden burden of NAFLD in this high-risk group. While this may have resulted in a slight underestimation of the true prevalence, the small number of known NAFLD diagnoses in our cohort suggests that the impact on the overall prevalence estimates is minimal. Lastly, the study was conducted at a single center, which could limit the generalizability of our findings. However, as a tertiary referral hospital, JUH receives patients from all over Jordan, making our sample reasonably representative of the broader population. Nevertheless, further multicenter, prospective studies with larger sample sizes are needed to provide a more comprehensive understanding of NAFLD prevalence in high-risk patients and to refine the most effective screening strategies.

## 5. Conclusion

Undiagnosed NAFLD and liver fibrosis prevalence is high, supporting ADA guidelines for universal screening in T2D and prediabetes. Due to discrepancies among screening systems, we recommend using multiple systems or developing a new one that integrates the current elements and considers sex, BMI, and insulin resistance.

## Figures and Tables

**Table 1:** Patients' characteristics.

		Count	%	Mean	Standard deviation
Sex	Male	94	37		
	Female	160	63		

Age				59.15	10.09

BMI				34.14	6.60

Waist circumference (cm)				111.29	14.39

DM 2	No	36	14.2		
	Yes	218	85.8		

Pre-diabetes	No	218	85.8		
	Yes	36	14.2		

Duration of diabetes (years)				10.56	9.09

Metformin	No	47	18.9		
	Yes	202	81.1		

Insulin	No	145	60.4		
	Yes	95	39.6		

**Table 2:** Patient characteristics identified with fibrosis according to FIB-4 score.

		None to mild fibrosis (< 1.3)				Moderate–severe fibrosis (≥ 1.3)			
		Count	%	Mean	SD	Count	%	Mean	SD
Gender	Male	67	75.3			22	24.7		
	Female	124	81.6			28	18.4		

Age^∗^				56.72	9.11			67.36	9.44

BMI				34.41	6.61			32.79	6.79

Waist circumference (cm)				110.99	14.37			111.70	14.62

Diabetes	No	27	77.1			8	22.9		
	Yes	165	79.7			42	20.3		

Prediabetes	No	165	79.7			42	20.3		
	Yes	27	77.1			8	22.9		

Duration of diabetes^∗^ (years)				9.64	8.81			12.63	9.18

Metformin^∗^	No	29	67.4			14	32.6		
	Yes	160	82.5			34	17.5		

Insulin	No	112	80.0			28	20.0		
	Yes	69	77.5			20	22.5		

Abbreviation: SD, standard deviation.

^∗^Statistically significant correlation.

^∗^ *p* < 0.05.

**Table 3:** Patient characteristics identified with fibrosis according to NFS (excluding indeterminate cases).

		None to mild fibrosis (F0–F2)				Moderate–severe fibrosis (F3-F4)			
		Count	%	Mean	SD	Count	%	Mean	SD
Gender	Male	21	61.8			13	38.2		
	Female	43	68.3			20	31.7		

Age^∗^				51.33	8.18			68.06	8.21

BMI^∗^				31.46	4.36			37.96	7.55

Waist circumference^∗^ (cm)				103.30	9.49			118.02	12.26

Diabetes	No	12	63.2			7	36.8		
	Yes	52	66.7			26	33.3		

Prediabetes	No	52	66.7			26	33.3		
	Yes	12	63.2			7	36.8		

Duration of diabetes^∗^ (years)				6.03	6.06			12.84	10.31

Metformin	No	6	33.3			12	66.7		
	Yes	58	77.3			17	22.7		

Insulin	No	45	72.6			17	27.4		
	Yes	17	53.1			15	46.9		

Abbreviation: SD, standard deviation.

^∗^Statistically significant correlation.

^∗^ *p* < 0.05.

**Table 4:** Patient characteristics identified with NAFLD according to FLI score (excluding indeterminate cases).

		No steatosis (< 20)				Probable steatosis (≥ 60)			
		Count	%	Mean	SD	Count	%	Mean	SD
Gender	Male	15	18.5			66	81.5		
	Female	37	25.7			107	74.3		

Age				59.29	10.96			59.43	9.57

BMI^∗^				32.40	7.23			35.55	6.23

Waist circumference (cm)^∗^				107.40	19.08			114.70	12.03

Diabetes	No	10	31.3			22	68.8		
	Yes	42	21.6			152	78.4		

Prediabetes	No	42	21.6			152	78.4		
	Yes	10	31.3			22	68.8		

Duration of diabetes (years)				8.72	8.42			11.31	9.49

Metformin^∗^	No	10	23.3			33	76.7		
	Yes	40	22.5			138	77.5		

Insulin	No	33	25.2			98	74.8		
	Yes	15	18.5			66	81.5		

*Note:* ^∗^ *p* < 0.05 = 0.000, waist circumference = 0.001, and BMI = 0.006.

Abbreviation: SD, standard deviation.

^∗^Statistically significant correlation.

**Table 5:** Discrepancy between the scoring systems.

			FIB-4	
			None to mild fibrosis	Moderate–severe fibrosis
NFS	None to mild fibrosis	Count	63	26
		%	87.6%	12.4%
	Moderate–severe fibrosis	Count	9	24
		%	27.3%	72.7%

FLI	No steatosis	Count	40	11
		%	78.4%	21.6%
	Probable steatosis	Count	133	30
		%	81.6%	18.4%

**Table 6:** Factors associated with discrepancy between FIB-4 and other scoring systems.

		FIB-4 and FLI				FIB-4 and NFS			
		No		Yes		No		Yes	
		Mean (%)	SD	Mean (%)	SD	Mean (%)	SD	Mean (%)	SD
Gender	Male	98.3%^∗^		1.7%		89.7%		10.3%	
	Female	89.8%		10.2%		80.6%		19.4%	

Age		59	10.33	58	9.91	59	10.62	60	8.44

BMI		34.09	7.23	37.85	5.66	33.59^∗^	6.82	38.38	7.78

Waist circumference (cm)		111.3	15.56	115.5	18.98	111.0	15.38	115.0	17.79

Diabetes	No	78.3%		21.7%		65.2%		34.8%	
	Yes	95.5%^∗^		4.5%		87.2%^∗^		12.8%	

Prediabetes	No	95.5%		4.5%		87.2%		12.8%	
	Yes	78.3%		21.7%		65.2%		34.8%	

Duration of diabetes		10.81	8.46	8.91	10.94	11.22	8.50		8.91

Metformin	No	90.5%		9.5%		85.7%		14.3%	
	Yes	93.1%		6.9%		83.1%		16.9%	

Insulin	No	94.3%		5.7%		85.2%		14.8%	
	Yes	92.9%		7.1%		82.1%		17.9%	

Abbreviation: SD, standard deviation.

^∗^Statistically significant correlation.

^∗^ *p* < 0.05.

## Data Availability

The data supporting the findings of this study are available from the corresponding author upon request.
